# TESTING HEALTH DISPARITIES IN COGNITIVE AND BIOLOGICAL AGING IN OLDER ADULTS IN THE UNITED STATES

**DOI:** 10.1093/geroni/igz038.1595

**Published:** 2019-11-08

**Authors:** Daniel Belsky

**Affiliations:** Columbia University Mailman School of Public Health, New York, New York, United States

## Abstract

We conducted analysis to test if health disparities in cognitive aging were parallel to or different from health disparities in patterns of aging in other systems in the body, and if race/ethnicity-related disparities could be accounted for by differences in socioeconomic circumstances across the life-course. We analyzed data from more than 10,000 adults participating in the US NHANES and US Health and Retirement Study. We measured cognitive aging using neuropsychological tests of processing speed and memory. We measured aging in other systems using composite indices of biological aging based on organ-system function tests and blood chemistries. We conducted analysis to (i) quantify and compare health disparities in cognitive aging and biological aging; (ii) test if individuals exhibiting accelerated cognitive aging were also exhibiting accelerated biological aging; and (iii) test if race/ethnic disparities in cognitive and biological aging could be explained by measured socioeconomic resource differences in childhood and later life.

